# Decreased mean platelet volume predicts poor prognosis in invasive bladder cancer

**DOI:** 10.18632/oncotarget.19242

**Published:** 2017-07-12

**Authors:** Xin Wang, Ming-Ming Cui, Yangyang Xu, Li Liu, Ye Niu, Tiemin Liu, Zhi-Ping Liu, Rui-Tao Wang, Kai-Jiang Yu

**Affiliations:** ^1^ Department of Internal Medicine, Harbin Medical University Cancer Hospital, Harbin Medical University, Harbin, Heilongjiang 150081, China; ^2^ Department of Urinary Surgery, Harbin Medical University Cancer Hospital, Harbin Medical University, Harbin, Heilongjiang 150081, China; ^3^ Heilongjiang Academy of Medical Science, Harbin, Heilongjiang 150081, China; ^4^ Department of Geriatrics, The Second Affiliated Hospital, Harbin Medical University, Harbin, Heilongjiang 150086, China; ^5^ Division of Hypothalamic Research, Department of Internal Medicine, UT Southwestern Medical Center, Dallas, TX 75390, USA; ^6^ Department of Internal Medicine, University of Texas Southwestern Medical Center, Dallas, TX 75390, USA; ^7^ Department of Intensive Care Unit, Harbin Medical University Cancer Hospital, Harbin Medical University, Harbin, Heilongjiang 150081, China

**Keywords:** bladder cancer, mean platelet volume, prognosis

## Abstract

**Background:**

Altered mean platelet volume (MPV) is implicated in a wide range of cancers. However, the prognostic role of MPV in muscle-invasive bladder cancer (MIBC) remains largely unknown. The purpose of this study was to elucidate the predictive significance of MPV in MIBC.

**Method:**

The retrospective study included 218 consecutive MIBC patients between January 2009 and December 2009. The relationships between MPV and clinicopathological characteristics were analyzed. Kaplan-Meier method and Cox regression were used to evaluate the prognostic impact of MPV.

**Result:**

Of the 218 patients, low MPV levels were detected in 141 (64.7 %) patients. Reduced MPV was associated with T stage and histology grade (p < 0.05). In the Kaplan-Meier analysis, decreased MPV was significantly associated with a poorer overall survival (p = 0.007). In the multivariate Cox model, decreased MPV was an independent prognostic index for overall survival (HR=2.023, 95% CI=1.050-3.897, p = 0.025).

**Conclusion:**

MPV is easily available in routine blood test. Our results demonstrated that reduced MPV could be regarded as a potential prognosis indicator for clinical outcome in MIBC.

## INTRODUCTION

Bladder cancer (BC) is one of the most frequently diagnosed malignancies and is a common cause of cancer-related mortality worldwide. Approximately 25% of the bladder cancers are found to be muscle invasive (MIBC) with a 5-year survival proportion of 33% [1]. Therefore, developing appropriate and effective biomarkers to predict clinical outcome is crucial for elucidating the mechanism underlying metastasis and recurrence in MIBC, resulting in discovery of novel therapeutic agents.

Platelets play a pivotal role in cancer progression and metastasis. There is emerging evidence to suggest that platelets mediate tumor cell growth, angiogenesis, and dissemination [2]. Increased platelets are correlated with a decrease in overall survival and poorer prognosis in various types of cancer, including pancreatic cancer, gastric cancer, colorectal cancer, endometrial cancer, and ovarian cancer [3–7]. However, platelet count is determined by the balance between the rate of production and consumption of platelets. A normal platelet count could conceal the presence of highly hypercoagulative and pro-inflammatory cancer phenotypes in the presence of efficient compensatory mechanisms [8].

Mean platelet volume (MPV), the most commonly used measure of platelet size, is a surrogate marker of platelet activation [9]. Altered MPV levels were found in gastric cancer, ovarian cancer, lung cancer, colon cancer, and breast cancer [10–14]. In addition, MPV is associated with the prognosis in patients with non-small cell lung cancer and multiple myeloma [15, 16]. However, its clinical implications in MIBC have not been well defined. Therefore, the purpose of the current study was to evaluate the association of MPV levels with prognosis in MIBC cases.

## RESULTS

Between Jan, 2009 and Dec, 2009, a total of 218 MIBC patients were enrolled in this study. Among the 218 patients, 46 (21.1) were women and 172 (78.9) were men, and the median age was 63.2 ± 9.0 years (range 31-82).

**Figure 1:**
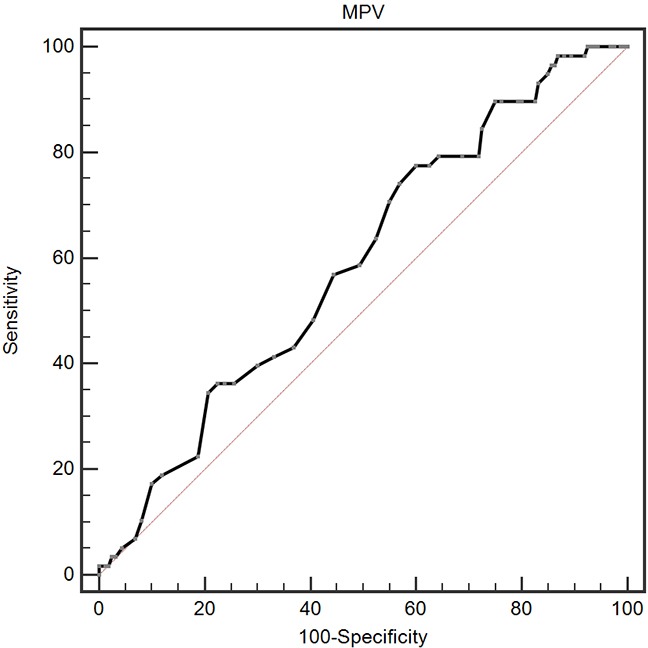
Optimized cut-off value was determined for MPV using standard ROC curve analysis

A ROC curve for OS prediction was plotted to verify the optimal cut-off value for MPV, which was 9.1 fL (Figure 1). It demonstrated that MPV predicts cancer prognosis with a sensitivity of 77.6 % and a specificity of 40.0 % (AUC = 0.591, 95 % CI: 0.522-0.657, p = 0.031). Then, patients were divided into 2 groups: patients with MPV ≤ 9.1 fL and patients with MPV > 9.1 fL. There were 141 (64.7 %) patients with MPV ≤ 9.1 fL and 77 (35.3 %) patients with MPV > 9.1 fL.

The relationships between MPV and clinical characteristics were shown in Tables 1 and 2. Our study revealed that MPV was associated with T stage and histology grade. However, no significant differences were found between the groups with regard to age, gender, multiplicity, growth pattern, distant metastasis, and lymphovascular invasion.

**Table 1:** Baseline characteristics of the patients according to MPV levels

Variables	Total	MPV > 9.1	MPV ≤ 9.1	P value
	n (%)	n (%)	n (%)	
Age (years)				0.657
≤ 65	123 (56.4)	45 (58.4)	78 (55.3)	
> 65	95 (43.6)	32 (41.6)	63 (44.7)	
Gender				0.665
Male	172 (78.9)	62 (80.5)	110 (78.0)	
Female	46 (21.1)	15 (19.5)	31 (22.0)	
T stage				< 0.001
T2	144 (66.1)	53 (9.2)	91 (19.2)	
T3	26 (11.9)	11 (50.4)	15 (50.4)	
T4	48 (22.0)	13 (40.4)	35 (30.4)	
Histology grade				0.028
G1+G2	114 (52.3)	48 (62.3)	66 (46.8)	
G3	104 (47.7)	29 (37.7)	75 (53.2)	
Multiplicity				0.474
Solitary	126 (57.8)	47 (61.0)	79 (56.0)	
Multifocal	92 (42.2)	30 (39.0)	62 (44.0)	
Growth pattern				0.699
Papillary	84 (38.5)	31 (40.3)	53 (37.6)	
Non-papillary	134 (61.5)	46 (59.7)	88 (62.4)	
Lymphovascular invasion				0.081
Yes	82 (37.6)	23 (29.9)	59 (41.8)	
No	136 (62.4)	54 (70.1)	82 (58.2)	

MPV, mean platelet volume.

**Table 2:** Baseline characteristics of the patients according to MPV levels

Variables	MPV > 9.1	MPV ≤ 9.1	P value
Age (years)	62.2 (9.5)	63.8 (8.7)	0.189
Gender (male, %)	62 (80.5)	110 (78.0)	0.665
Smoker (n, %)	15 (19.5)	23 (16.3)	0.556
Drinking (n, %)	8 (10.4)	16 (11.3)	0.829
BMI (kg/m^2^)	23.4(3.0)	23.5 (3.1)	0.781
FPG (mmol/L)	5.10 (4.80-5.55)	5.00 (4.70-5.30)	0.040
WBC (×10^9^/L)	7.25 (2.87)	7.06 (2.53)	0.615
Neutrophils (×10^9^/L)	4.54 (2.49)	4.60 (2.35)	0.876
Lymphocytes (×10^9^/L)	2.03 (0.71)	1.80 (0.56)	0.017
Hemoglobin (g/dl)	138.2 (17.0)	133.5 (18.4)	0.068
Platelet count (×10^9^/L)	199.2 (51.1)	223.1 (65.7)	0.003
PDW (%)	17.2 (1.2)	16.7 (0.8)	< 0.001
NLR	2.80 (2.67)	2.70 (2.17)	0.760
PLR	119.1 (58.3)	128.3 (52.6)	0.238

Data are expressed as means (SD) or median (IQR). FPG, fasting plasma glucose; WBC, white blood cell; BMI, body mass index; MPV, mean platelet volume; NLR, neutrophil-to-lymphocyte ratio; PLR, platelet-to-lymphocyte ratio.

**Figure 2:**
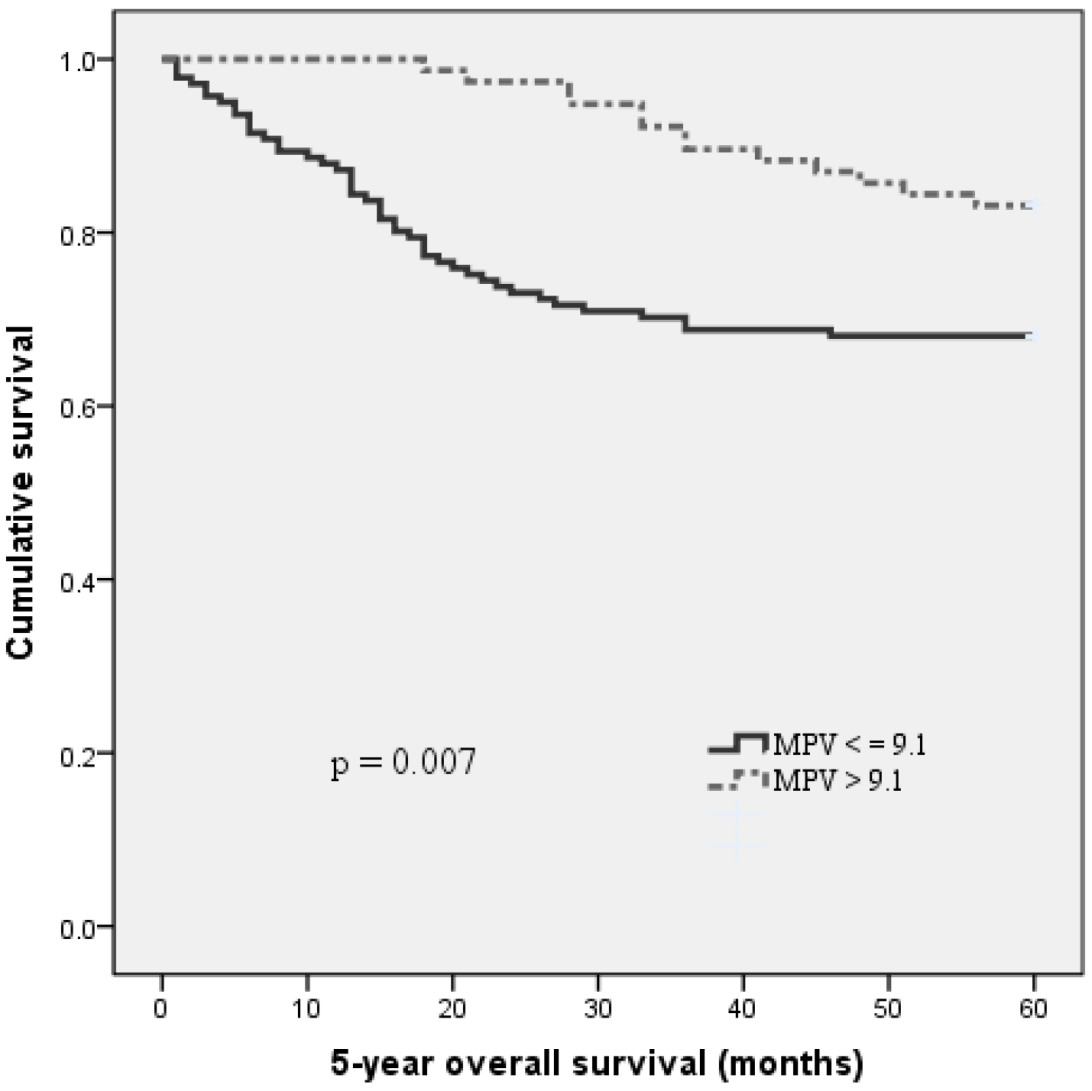
Kaplan–Meier analysis of overall survival in MIBC patients

With a median follow up of 60 months, 58 (26.6 %) patients had death events. Patients with MPV ≤ 9.1 fL had a significantly shorter 5-year OS than patients with MPV > 9.1 fL (68.1 % vs. 83.1 %, p = 0.007). The Kaplan-Meier OS curves of the normal versus reduced MPV showed a significant separation (Figure 2).

In univariate analysis, age (categorical variable), MPV (categorical variable), histology grade, lymphovascular invasion, multiplicity, and growth pattern were significant predictors of OS (Table 3). NLR (p = 0.060), PLR (p = 0.070) and T stage (p = 0.059) showed weak associations. Other parameters were not found to be in correlation with OS. Next, all the factors with a P value less than 0.10 in univariate analysis were included in multivariate analysis (Table 4). In multivariate analyses, we demonstrated that MPV was an independent prognostic factor in patients with MIBC (hazard ratio (HR): 2.023 [95% confidence interval (CI): 1.050-3.897, p =0.025]).

**Table 3:** Result of the univariate analysis of overall survival in patients with bladder cancer

	Hazard ratio	95% CI	P-value
Age (years) (≥ 65 versus < 65)	2.263	1.336-3.831	0.002
Gender (male versus female)	0.569	0.270-1.200	0.138
Smoker (yes versus no)	1.019	0.515-2.014	0.957
BMI (kg/m^2^)	0.957	0.879-1.042	0.311
FPG (mmol/L)	0.879	0.564-1.370	0.569
WBC (×10^9^/L)	1.064	0.978-1.158	0.148
Hemoglobin (g/dl)	1.002	0.987-1.018	0.778
Platelet count (×10^9^/L)	1.000	0.996-1.004	0.945
MPV (fL) (≤ 9.1 versus > 9.1)	2.274	1.226-4.217	0.009
PDW (%)	0.833	0.631-1.101	0.199
NLR	1.082	0.997-1.175	0.060
PLR	1.004	1.000-1.008	0.070
Histology Grade			
(G3 versus G1+G2)	3.482	1.995-6.078	< 0.001
Lymphovascular invasion			
(Yes versus No)	3.262	1.925-5.527	< 0.001
Multiplicity			
(Multifocal versus Solitary)	0.696	0.405-1.196	0.189
Growth Pattern			
(Papillary versus Non-papillary)	0.374	0.198-0.706	0.002
T stage			
(T3+T4 versus T2)	1.648	0.982-2.765	0.059
Chemotherapy (yes versus no)	0.802	0.380-1.691	0.562

**Abbreviations:** see to Tables 1 and 2.

**Table 4:** Result of the multivariate analysis of overall survival in patients with bladder cancer

	Hazard ratio	95% CI	P-value
Age (years) (≥ 65 versus < 65)	1.785	1.037-3.074	0.031
MPV (fL) (≤ 9.1 versus > 9.1)	2.023	1.050-3.897	0.025
NLR	1.000	0.994-1.007	0.892
PLR	1.062	0.923-1.223	0.402
Histology Grade			
(G3 versus G1+G2)	3.404	1.902-6.090	< 0.001
Lymphovascular invasion			
(Yes versus No)	3.272	1.805-5.933	< 0.001
Growth Pattern			
(Papillary versus Non-papillary)	0.419	0.208-0.841	0.014
T stage			
(T3+T4 versus T2)	0.546	0.288-1.037	0.064

CI, confidence interval. Abbreviations: see to Tables 1 and 2.

## DISCUSSION

This study found that MPV is correlated with patient’s survival and is an independent risk factor for prognosis. Our findings suggested the potential importance of assessing bladder cancer prognosis by combining clinicopathological characteristics with platelet index.

Thrombocytosis is associated with reduced survival in several cancers, such as lung cancer, ovary cancer, endometrium cancer, rectum cancer, kidney cancer, gastric cancer, pancreas cancer, and breast cancer. Increased platelets facilitate cancer progression and metastasis by promoting angiogenesis and tumor cell establishment at distant sites [17]. For bladder cancer, elevated expression of platelet-derived endothelial cell growth factor is markedly correlated with the tumor progression of bladder cancer [18]. Moreover, platelet-derived growth factor receptor beta is found to be a biomarker for predicting NMIBC recurrence [19]. However, these indices for reflecting activated platelets were expensive and not commonly evaluated in the clinical setting. In accordance with the studies above, the present study indirectly confirmed the findings using a simple marker of platelet activation.

Inflammation may be responsible for the association between MPV and survival. There is a strong linkage between inflammation and cancer [20]. Moreover, platelet plays an essential role in inflammation and cancer. MPV is an early parameter of activated platelets. Large platelets are more reactive than their smaller counterparts in releasing a variety of pro-inflammatory cytokines, and are more likely to aggregate. The aggregation at sites of inflammation leads to the intensive infiltration of large platelets into vascular and intestinal wall, and the reduction of platelet size [21]. On the other hand, the release rate of small size platelets from the bone marrow increased since excessive pro-inflammatory cytokines interfere with megakaryopoiesis [22]. Therefore, lower MPV values could be suggestive of an enhanced consumption of large platelets in inflammatory states [9]. Recent reports confirmed that low levels of MPV are associated with high-grade inflammatory diseases and reverse in the course of anti-inflammatory therapy [9].

In agreement with our results, Inagaki N and Kumagai S *et al* found that low MPV level was associated with poor prognosis in non-small-cell lung cancer [12, 23]. Aksoy *et al* reported that solid tumors with bone marrow metastasis were more likely to have low MPV levels [24]. These data are also consistent with the current knowledge that anti-platelet is considered to be a part of cancer adjuvant therapy [2].

This is the first time to investigate the correlation between MPV and clinicopathologic parameters, as well as prognosis in patients with MIBC. In addition, we established platelet activation in MIBC using an inexpensive laboratory indicator. Further experiments are needed to elucidate the underlying mechanism. Activated platelets may be considered as an excellent therapeutic target for MIBC patients. However, there are several limitations in our study. First, this study was retrospective and further prospective studies are needed to confirm and update our conclusion. Second, we were unable to explore the exact mechanism of decreased MPV in bladder cancer. Third, the cases were composed of Chinese. The application to other ethnic groups still needs further investigation.

In conclusion, reduced MPV has prognostic value in MIBC patients. Future studies need to be focused on the underlying mechanisms of MPV in MIBC.

## MATERIALS AND METHODS

### Study population

This study consisted of 218 consecutive MIBC cases (mean age 63.2 ± 9.0 years, range 31-82 years). Cases were admitted to Harbin Medical University Cancer Hospital, Harbin Medical University between January 2009 and December 2009. All patients undergone radical cystectomy. The pathologic diagnoses of MIBC were evaluated by pathologists from biopsy reports. None of the patients received preoperative chemotherapy or radiation therapy. Patients were excluded if they had hematological disorders, coronary artery disease, hypertension, diabetes mellitus, and medical treatment with anticoagulant, statins, and acetylic salicylic acid. Standard demographic and clinicopathological data were collected from the patients’ records in hospital. Survival data were obtained through follow-up. Overall survival (OS) was defined as the interval from the date of diagnosis to death or last follow-up. The median follow-up time was 60 months.

Venous blood samples after an 10-hour overnight fasting were collected from the individuals within 1 week prior to surgery. White blood cell (WBC), haemoglobin, and platelet indices were measured by an autoanalyzer (Sysmex XE-2100, Kobe, Japan). The whole blood samples were collected in EDTA-containing tubes, and all samples were processed within 30 minutes after blood collection. The platelet-to-lymphocyte ratio (PLR) was calculated as the absolute platelet count measured in × 10^9^/L divided by the absolute lymphocyte count measured in ×10^9^/L. The neutrophil-to-lymphocyte ratio (NLR) was calculated as the absolute neutrophil count measured in × 10^9^/L divided by the absolute lymphocyte count measured in × 10^9^/L.

The Institutional Ethics Review Board of Harbin Medical University Cancer Hospital of Harbin Medical University approved this study prior to commencement of data collection and waived the informed consent requirement because it was a retrospective study.

### Statistical analysis

All continuous data are expressed as means ± SD or medians (interquartile range), and the categorical data are expressed in percentages. The continuous variables were compared with Student’s t test or the Mann-Whitney U test, as appropriate, whereas categorical variables were compared with the Chi-square test. The Kaplan-Meier method was used for descriptive analysis of survival curves; survival curves were compared using log-rank tests. We used the univariate Cox proportional hazards model for identifying the contribution of each variables. The multivariate Cox proportional hazards models were used to determine adjusted hazard ratios for survival. Variables with P-values < 0.1 were selected for the multivariate analysis. Receiver-operating characteristics (ROC) curve analysis was performed to identify cut-off value of MPV. P < 0.05 was considered to indicate statistically significant differences in all tests. All statistical analyses were performed using SPSS Statistics version 22.0 (SPSS Inc., Chicago, IL, USA).
